# EGF-Like-Domain-7 Is Required for VEGF-Induced Akt/ERK Activation and Vascular Tube Formation in an *Ex Vivo* Angiogenesis Assay

**DOI:** 10.1371/journal.pone.0091849

**Published:** 2014-03-19

**Authors:** Kimio Takeuchi, Ryoji Yanai, Fumiaki Kumase, Yuki Morizane, Jun Suzuki, Maki Kayama, Katarzyna Brodowska, Mitsuru Nakazawa, Joan W. Miller, Kip M. Connor, Demetrios G. Vavvas

**Affiliations:** 1 The Retina Service, Angiogenesis Laboratory, Massachusetts Eye and Ear Infirmary, Department of Ophthalmology, Harvard Medical School, Boston, Massachusetts, United States of America; 2 Department of Ophthalmology, Okayama University Graduate School of Medicine, Dentistry and Pharmaceutical Sciences, Okayama, Japan; 3 Department of Ophthalmology, Hirosaki University Graduate School of Medicine, Aomori, Japan; University of Patras, Greece

## Abstract

EGFL7 is a secreted angiogenic factor, which in contrast to the well-known secreted angiogenic molecules VEGF and FGF-2, is almost exclusively expressed by endothelial cells and may act in an autocrine fashion. Prior studies have shown EGFL7 to mediate its angiogenic effects by interfering with the Notch pathway and/or via the intronic miR126. Less is known about its effects on VEGF signaling. We wanted to investigate the role of epidermal growth factor-like domain 7 (EGFL7) in VEGF-driven angiogenesis using an *ex vivo* Matrigel-embedded mouse eye cup assay and siRNA mediated knockdown of EGFL7 by siRNA. Our results suggested that VEGF-induced vascular tube formation was significantly impaired after siRNA downregulation of EGFL7. In addition, knockdown of EGFL7 suppressed VEGF upregulation of phospho-Akt and phospho-Erk(1/2) in endothelial cells, but did not alter VEGFR phosphorylation and neuropilin-1 protein expression or miR126 expression. Thus, in conclusion, EGFL7 is required for VEGF upregulation of the Akt/Erk (1/2) pathway during angiogenesis, and may represent a new therapeutic target in diseases of pathological neovascularization.

## Introduction

Angiogenesis is an important biological process not only under physiological conditions, but also in a variety of diseases including cancer, rheumatoid arthritis [1]–[4], age-related macular degeneration [5], diabetic retinopathy [6], retinal vein occlusion [7], and retinopathy of prematurity [8]. It is fundamental in many biological processes including development, reproduction and wound repair. With the exception of the vasculature of the female reproductive system, the endothelium of the adult vasculature is normally quiescent. The vasculature becomes activated and grows new capillaries through angiogenesis in response to appropriate stimuli (i.e., injury, atherosclerosis, tumor growth and metastasis, arthritis). Under these conditions, angiogenesis is a highly regulated process. The sprouting of vessels involves activation of quiescent endothelial cells, proteolytic degradation of the extracellular matrix, chemotactic migration, invasion into the surrounding stroma, and endothelial cell proliferation and differentiation [2], [9]–[12]. Numerous inducers of angiogenesis have been identified, including the members of the vascular endothelial growth factor (VEGF) family, angiopoietins, transforming growth factors (TGFs), platelet-derived growth factor (PDGF), tumor necrosis factor alpha (TNF-α), interleukins, and members of the fibroblast growth factor (FGF) family [13], [14].

Recently, the novel angiogenic factor epidermal growth factor (EGF)-like domain 7 (EGFL7) has been described [15]. EGFL7 is a 41-kDa secreted angiogenic factor with high homology among vertebrates. In unique contrast to the well-known secreted angiogenic molecules VEGF and FGF-2, which are mainly produced by non-endothelial cells, EGFL7 is almost exclusively expressed by endothelial cells and may act in an autocrine fashion [16]–[19]. It is expressed at high levels early during mouse embryonic development and is strictly associated with the vascular bed. Prior studies have shown EGFL7 to mediate its angiogenic effects by interfering with the Notch pathway [20], [21]. However, the role of *Egfl7* in vascular development has been complicated by the presence of the microRNA miR126 within its gene. Thus, knockout studies of EGFL7 may alter the epigenetic regulation of angiogenesis by miR126. When Kuhnert et al. [22] generated an EGFL7 knockout that preserved miR126 expression, they did not observe vascular abnormalities, casting doubt on the role of EGFL7 in vascular development. Kuhnert et al. went as far as stating that the observed phenotype in the prior studies was due to miR126 deregulation since in their study, targeted disruption of miR126 (but not EGFL7) led to phenotypic changes seen in the prior non-specific EGFL7-knockdown animals. However, in zebrafish, morpholino antisense oligonucleotides targeting *egfl7* resulted in vascular defects that were rescued by co-injection of *egfl7* mRNA [18]; this indicates that EGFL7 has a defined function (at least in zebrafish vascular development) that is not being compensated by other genes [18].

Thus, given that the mouse *Egfl7* loss-of-function phenotype and mechanism of action is still unclear, we wanted to investigate the role EGFL7 in VEGF tube formation and signaling using an *ex vivo* angiogenesis assay.

## Materials and Methods

### Materials

CD31 antibody, which was used to stain the endothelial cells in Matrigel, was purchased from Novus Biologicals, Inc. (Littleton, CO). Secondary antibodies of Alexa Fluor 568 goat anti-rat IgG was purchased from Invitrogen (Carlsbad, CA). Growth Factor Reduced Matrigel™ Matrix (Phenol Red-free) and Cell Recovery Solution were purchased from BD Pharmingen (San Diego, CA). Mouse VEGF was purchased from R&D Systems (Minneapolis, MN). Oligofectamine and Opti-MEM were purchased from Life Technologies (Grand Island, NY). SiRNA targeting EGFL7 and control siRNA were purchased from Santa Cruz Biotechnology (Santa Cruz, CA).

### Matrigel Cultures of Freshly Cut Eye Tissue

Eyes were enucleated from 6- to 8-week-old C57BL/6 (*B6*) mice and rinsed in PBS (−) supplemented with 5 ml penicillin/streptomycin (100x) (Invitrogen, Carlsbad, CA). Eye samples (which contained retina-RPE-choroid-sclera) were excised in a circle around the optic nerve head using a 1.0-mm skin biopsy punch (Integra Miltex, Plainsboro, NJ) (Fig. S1). Freshly cut tissue samples were embedded in Matrigel and grown in 0.5 ml of Dulbecco’s modified Eagle’s medium (DMEM) (Life Technologies, Grand Island, NY) with 10% fetal bovine serum in a 24-well plate. For all experiments, cells were grown at 37°C in a humidified atmosphere of 5% CO_2_ and 95% air.

All animal experimental procedures were designed ethically to conform to both the ARVO Statement for the Use of Animals in Ophthalmic Vision Research and approved by the Institutional Animal Care and Use Committee (IACUC) of Massachusetts Eye and Ear Infirmary.

### Immunofluorescence Staining of Matrigel-embedded Mouse Eye Tissue

Immunofluorescence staining of Matrigel-embedded mouse eye tissue was performed according to Baker et al. [23]. Briefly, after the removal of the culture medium, Matrigel-embedded eye tissues samples were washed in PBS (+). Samples were then fixed in 4% PFA for 30 min at room temperature, and permeabilized in PBS(+) supplemented with 0.25% Triton X-100 two times at room temperature. Nonspecific antibody binding was blocked with 5% BSA in PBS (+) supplemented with 0.1 ml of 1 M MnCl2 and 1% Tween-20. Samples were incubated overnight with rat monoclonal CD31 antibody, 1: 1000 dilution at 4°C. Then, after washing three times with PBS(−) supplemented with 0.1% Triton X-100, samples were incubated for 2 hours with Alexa Fluor 568 goat anti-rat IgG. Samples were then rinsed three times in PBS(−) before mounting, and images were acquired with Zeiss Axioplan microscope.

### siRNA Transfection

Small interfering RNA (siRNA) transfection was performed using Oligofectamine according to the protocol of Baker et al. [23]. After the preparation of solution “A” (2.5 μl of 40 μM siRNA stock in 182.5 μl of Opti-MEM per sample) and solution “B” (3 μl of Oligofectamine reagent in 12 μl of Opti-MEM per sample), respectively, solution A and B were mixed and placed for 20 min at RT beforehand. And, after Matrigel-embedded mouse eye tissue of each well was filled with 800 μl Opti-MEM in 24-well plate, 200 μl of A+B mixed solution was added to each well to achieve a final volume of 1 ml. Each sample was incubated overnight at 37°C and 5% CO_2_ in the Opti-MEM mixture.

### Preparation of Antibody-coated Magnetic Beads for Endothelial Cell Isolation

Dynabeads sheep anti-rat IgG was washed three times with PBS containing 0.1% bovine serum albumin (BSA) and then incubated with rat anti-mouse CD31 monoclonal antibody for 2 hours at room temperature. Following incubation, beads were washed three times with PBS containing 0.1% BSA and resuspended in the same medium.

### Isolation of Endothelial Cells

Matrigel-embedded eye tissue samples were treated with Cell Recovery Solution supplemented with phosphatase and protease inhibitor mixture for 15 minuts at 4°C, and cells were isolated using anti CD31-coated Dynabeads.

### Real-time Quantitative RT-PCR (qRT-PCR)

Total RNA was harvested from isolated cells using the RNeasy kit (Qiagen, Valencia, CA), and complementary DNA (cDNA) was generated with oligo-dT primers and SuperScript Reverse III Transcriptase (Invitrogen) according to the manufacturer’s instructions. Real-time PCR was carried out using the following mouse TaqMan gene-expression assays (Applied Biosystems, Foster City, CA): CD31 (Mm01242584_m1), α-SMA (Mm00725412_s1) and beta actin (Mm00607939_s1). All reactions were prepared following the manufacturer’s protocol and carried out using the StepOne™ Real-Time PCR System (Applied Biosystems).

### Protein Extraction and Western Blotting

Isolated cells were rinsed in ice-cold Tris-buffered saline and then lysed with lysis buffer (50 mM Tris-HCl, pH 7.6, 150 mM NaCl, 5 mM CaCl_2_, 1% Triton X-100 and 0.02% NaN3) supplemented with phosphatase and protease inhibitor mixture. For Western blotting, each group was collected from 12 to 20 of Matrigel-embedded eye samples. Cell suspensions were incubated on ice for 10 minutes and centrifuged at 14,000 rpm for 10 minutes at4°C. Supernatants were collected as whole-cell lysates. Protein concentration was determined by a DC protein assay kit (Bio-Rad, Hercules, CA). The proteins were separated by NuPAGE 4–12% Bis-Tris Gel (Life Technologies, Gaithersburg, MD) and transferred to a nitrocellulose membrane (Millipore, Bedford, MA). The blots were subsequently incubated with secondary antibodies conjugated to horseradish peroxidase (Cell Signaling Technologies). The membranes were then developed by ECL prime (Amersham, Piscataway, NJ, USA). Densitometric analysis of bands was performed using ImageJ software. Lane-loading differences were normalized by β-actin.

### Total RNA Extraction and miRNA Expression

To identify mir-126 expression in mouse endothelial cells after the treatment of EGFL7 siRNA, total RNA with mir-126 was extracted from mouse endothelial cells in the matrigel using QuantiGene Sample Processing Kit (Affymetrix, Santa Clara, CA) according to manufacture’s protocol. Following RNA isolation, miRNA expression was measured using QuantiGene 2.0 Reagent System (Affymetrix) according to manufacture’s protocol. To capture mir-126 from samples, the capture plates containing samples and working probe set (capture extender (CE), label extender (LE), blocking probe (BL)) were incubated overnight at 55°C±1°C for hybridization. After hybridization using the Pre-Amplifier and Amplifer, the capture plate was hybridized with the label probe according to manufacture’s protocol. Luminescence was measured using a microplate luminometer after adding of 2.0 Substrate according to manufacture’s protocol. The probe for miR126 sequence (ucguaccgugaguaauaaugcg) was purchased from Affymetrix. The number of each group is 4.

### Statistical Analysis

All experiments were repeated a minimum of three times. All data were expressed as means ± S.D. Statistical differences were analyzed by ANOVA test. Differences were considered significant at *P*<0.05.

## Results

### VEGF-induced Tube Formation is EGFL7 Dependent

**Figure 1:**
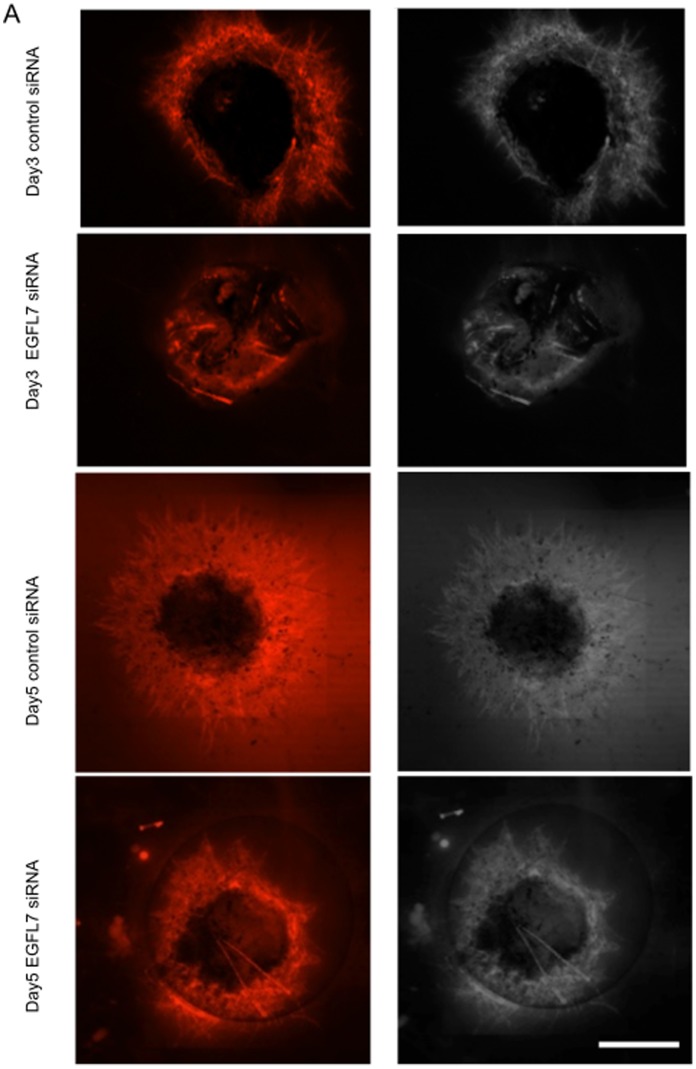
VEGF-induced tube formation is EGFL7 dependent. *A,* Mouse eye cups of each group were treated with EGFL7 or control siRNA after embedding them in Matrigel. Samples were cultured in VEGF (25 ng/ml) containing medium. At 3 and 5 days after knockdown of EGFL7, the tube length of neovascular from samples was evaluated by immunofluorescence using CD31 antibody. Bar equals 1000 μm. *B, ANOVA* Statistical analysis performed to evaluate the area of tube length. (n = 6) *, P<0.01. **, P<0.05. NS, not significant. C. At 3, 5 and 7 days after knockdown of EGFL7, endothelial cells were collected using anti-mouse CD31 antibody-coated magnetic beads (see Figure S4). The amounts of EGFL7 in isolated cells were examined by Western blotting.

Similar to aortic ring assay, VEGF application to the Matrigel-embedded mouse eye cup (Fig. S1) led to dose- and time-dependent neovessel tube formation from capillary-size vessels from mice eye cups (Fig. S2A–D). There was a rather linear response to VEGF from 12 ng/ml to 50 ng/ml. Neovessel formation was first seen at day 3 and continued to increase over a week (Fig. S2C–D). To investigate the role of EGFL7 in this process, loss-of-function experiments were performed using siRNA against EGFL7. As seen in Figure 1, siRNA knockdown of EGFL7 but not control scrambled siRNA resulted in significant decrease in tube formation both on day 3 and day 5. Since it is well known and mir-126 is localized within intron 7 of EGFL7 [24] and that some prior studies have shown that some of the angiogenic functions of EGFL7 maybe mediated via interference of its intronic miR126 we investigate whether EGFL7 knockdown by siRNA had any effects on the expression of mir-126 or not. As seen in Fig. S3, we could not observe a significant difference of miR-126 expression between control and EGFL7 siRNA treatment. This result indicates that EGFL7 knockdown by siRNA can inhibit angiogenesis independently of mir-126 levels.

### EGFL7 Knockdown does not Influence VEGFR2 Phosphorylation or Neuropilin 1 Expression

**Figure 2:**
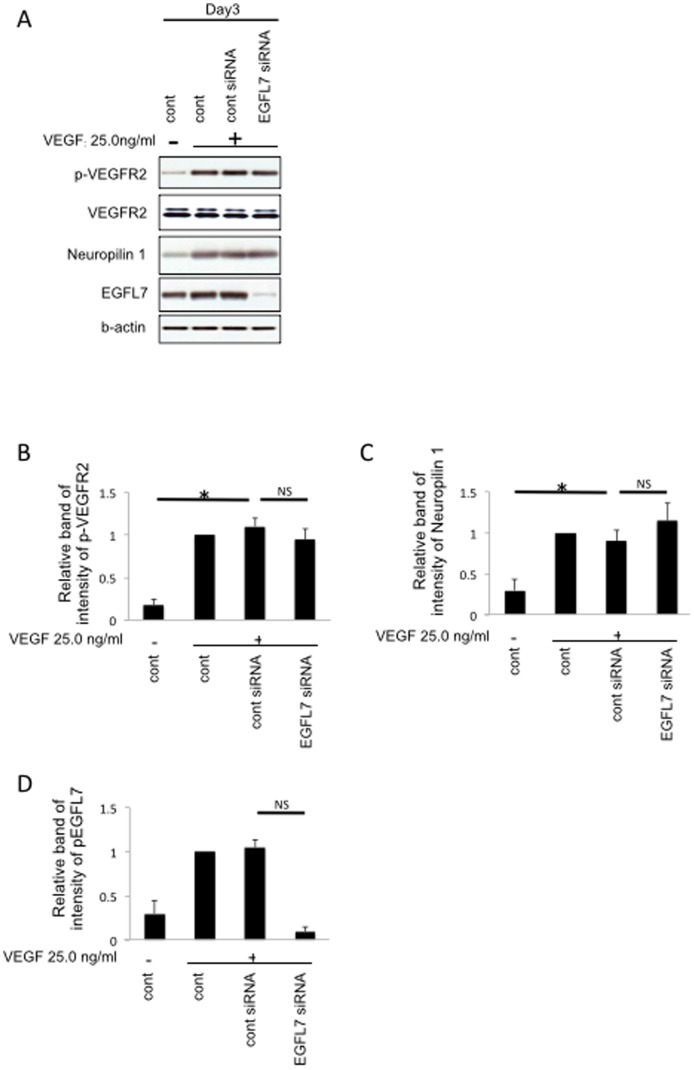
EGFL7 knockdown does not influence VEGFR2 phosphorylation or neuropilin 1 expression. *A,* Mouse eye cups of each group were treated with EGFL7 or control siRNA after embedding them in Matrigel. Samples were cultured in VEGF (25 ng/ml) containing medium. At 3 days after knockdown of EGFL7, endothelial cells were collected using anti-mouse CD31 antibody-coated magnetic beads (see Figure S4). The amounts of p-VEGFR2, neuropilin 1 and EGFL7 were examined by Western blotting. *B,* Densitometry of p-VEGFR2 in panel A. *C,* Densitometry of neuropilin 1 in panel A. *D,* Densitometry of EGFL7 in panel A. *ANOVA* Statistical analysis performed. (n = 3) *, P<0.01. **, P<0.05. NS, not significant. *, P<0.01. NS, not significant.

VEGF mediates its effects partially through VEGFR2 binding and phosphorylation as well as via Neuropilin 1 receptor that enhances VEGF binding to VEGFR2 by up to 6-fold [25]. In addition, it has been reported that VEGF can induce neuropilin 1 protein expression [26]. To investigate whether changes in VEGFR2 or neuropilin-1 expression after siRNA knockdown of EGFL7 are responsible for the observed effects, we purified endothelial cells from mouse eye cups using anti-mouse CD31 antibody-coated magnetic beads (Fig. S4 for enrichment and purity results) and analyzed the endothelial cells for VEGFR2 and neuropilin 1 via Western blotting. As seen in Fig. 2, EGFL7 knockdown did not influence VEGFR2 phosphorylation or Neuropilin 1 protein expression, suggesting that EGFL7 mediates its effects further downstream in VEGF signaling.

### EGFL7 Mediates VEGF-induced Activation of Akt and ERK(1/2)

**Figure 3:**
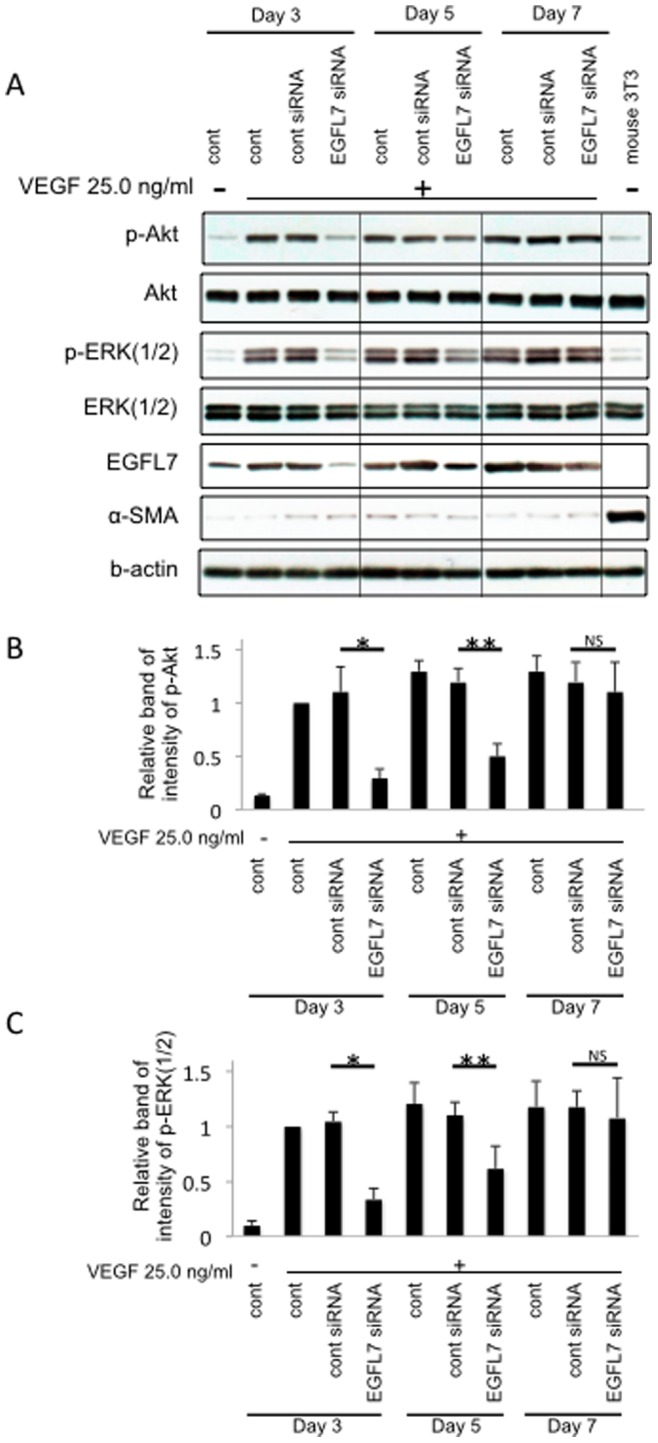
EGFL7 mediates VEGF-induced activation of Akt and ERK(1/2). *A,* Mouse eye cups of each group were treated with EGFL7 or control siRNA after embedding them in Matrigel. Samples were cultured in VEGF (25 ng/ml) containing medium. At 3, 5 and 7 days after knockdown of EGFL7, endothelial cells were collected using anti-mouse CD31 antibody-coated magnetic beads. The amounts of (p-)Akt, p-ERK(1/2), EGFL7 and α-SMA in isolated cells were examined by Western blotting. *B,* Densitometry of p-Akt in panel A. *C,* Densitometry of p-ERK(1/2) in panel A. *D,* Densitometry of EGFL7 in panel A. *ANOVA* Statistical analysis performed. (n = 3) *, P<0.01. **, P<0.05. NS, not significant. *, P<0.01. **, P<0.05. NS, not significant.

VEGF induced Akt and ERK activation are thought to be important mediators of VEGF-driven angiogenesis. To examine the role of EGFL7 in VEGF driven activation of Akt and ERK, siRNA knockdown experiments were performed. EGFL7 knockdown suppressed both phosphorylations of Akt and ERK(1/2) at day3 and day5 compared to the control siRNA (Fig. 3A,B and C). Moreover, as the effect of siRNA diminished with the passage of time (day 3→5→7), so did the effect on Akt and ERK(1/2) (Fig. 3A, D). These results suggest that EGFL7 is required for VEGF-induced Akt and ERK(1/2) phosphorylation during angiogenesis.

## Discussion

In the present study, we show that EGFL7 is required for the angiogenic effects of VEGF using a Matrigel-embedded mouse eye angiogenesis assay similar to the aortic ring assay and similar to the mouse eye cup described by Shao et al. [27]. Moreover, we showed that EGFL7 expression is needed for VEGF-induced upregulation of phospho-Akt and phospho-ERK(1/2) in endothelial cells for the first time.

EGFL7 is a novel secreted angiogenic factor with several key differences to the well-known secreted angiogenic molecules, VEGF and fibroblast growth factor-2, which are mainly produced by non-endothelial cells. In contrast, EGFL7 is almost exclusively expressed by the endothelial cells themselves [15]–[19], [28]. EGFL7 is expressed in a highly restricted manner in endothelial cells during embryonic development, when it plays a role in controlling the patterning and remodeling of vascular tubes during developmental vascularization [16], [17], [29], [30], and promotes angiogenesis [31]. EGFL7 expression markedly decreases in endothelial cells in postnatal life [16], consistent with a role in vascular development. However, the presence of miR126 within the *Egfl7* gene complicates investigations into its role in vascular development, because knockout studies of EGFL7 may alter epigenetic regulation by miR126 as well. When Kuhnert et al. [22] generated an *Egfl7* knockout that preserved miR126 expression, they did not observe vascular abnormalities, calling into question the role of EGFL7 in vascular development. They asserted that the observed phenotype in the prior studies was due to miR126 deregulation since targeted disruption of miR126, but not EGFL7, led to phenotypic changes seen in the prior nonspecific EGFL7-knockdown animals. However, in zebrafish, morpholino antisense oligonucleotides targeting egfl7 resulted in vascular defects that were rescued by co-injection of *egfl7* mRNA, indicating that *egfl7* has a defined function at least in zebrafish vascular development that is not compensated for by other genes [18]. Our study, using siRNA technology that targets the messenger RNA, bypasses interference with the intronic miR126 (see Fig. S3) and supports the notion that *egfl7* has direct angiogenic effects.

Prior studies have shown EGFL7 to mediate its angiogenic effects by interfering with the Notch pathway [20], [21]. In addition the EGFL7 intronic miR126 [32], [33] was shown to promote angiogenesis by inhibiting protein production of endogenous VEGF repressors within endothelial.

However, not much is known about the effects of EGFL7 on the VEGF/VEGFR-2/neuropilin 1 pathway. Neurolipin-1 exists on the cell membrane of endothelial cells as an isoform-specific receptor for VEGF and as a co-receptor of VEGFR-2. Though VEGF selectively up-regulates neuropilin 1 via the VEGFR-2 dependent pathway, Oh et al. indicated endothelial proliferation stimulated by VEGF was inhibited significantly by antibody perturbation of neuropilin 1, and selective neuropilin 1 inhibition suppressed neovascular formation substantially *in vivo* [26]. When we examined the effects of EGFL7 siRNA knockdown on VEGF signaling, we observed no significant effects on VEGFR2 phosphorylation or neuropilin 1 expression (Fig. 2). However, when we looked further downstream we found that EGFL7 was required for VEGF-induced activation of the Akt/Erk(1/2) pathways. Thus, EGFL7 not only has direct angiogenesis effects mediated by Notch signaling, but also indirect effects through the VEGF signaling pathway.

In conclusion, using the mouse eye cup *ex vivo* angiogenesis assay, we identified EGFL7 as required for VEGF-induced angiogenesis by facilitating VEGF-induced activation of Akt and Erk(1/2). EGFL7 may represent a target for diseases with pathological neovascularization.

## Supporting Information

Figure S1 **The creation of mouse eye cup and Histologic characterization of mouse eye cup embedded in Matrigel.** After the removal of mouse cornea and lens (A), the eye sample (which contains retina-RPE-choroid-sclera) was excised in a circle round the optic nerve head using 1.0-mm skin biopsy punch (B,C and D), and embedded it into Matrigel (E). Schema of posterior segment of the eye that is used (F). Endothelial cells on the frozen mouse eye tissue section were immunostained with anti-CD31 antibody (G.H). The colour was developed using HRP conjugated secondary antibody and DAB staining. The section was then counterstained with methyl green.
(TIFF)

Figure S2 **Dose and time dependent VEGF induction of neovascular tube formation from mouse eye cups.** *A,* After the eye cups were embedded in Matrigel, each concentration of VEGF (0, 12.5, 25.0, 50.0 ng/ml) was added in the medium. At 10 days after culturing in these concentrations of VEGF-containing medium, the area of neovascularization from samples was evaluated by immunofluorescence using CD31 antibody. Medium was changed at days 3 and 7 after embedding. Bar equals 1000 μm. *B, ANOVA* Statistical analysis performed to evaluate the area of tube length (n = 6). *, P<0.01. C, After the eye tissue samples were embedded in Matrigel, tissue samples were cultured in medium containing 25.0 ng/ml VEGF for 3, 7, or 10 days,. At each day after culturing in VEGF-containing medium, the area of neovascular from samples was evaluated by immunofluorescence using CD31 antibody. Medium was changed at day 3 and 7 after embedding. Bar equals 1000 μm. *D,* ANOVA Statistical analysis performed to evaluate the area of tube length (n = 6). *, P<0.01. **, P<0.05.
(TIFF)

Figure S3 **To identify mir-126 expression in mouse endothelial cells after the treatment of EGFL7 siRNA, total RNA with mir-126 was extracted from mouse endothelial cells in the matrigel using QuantiGene Sample Processing Kit (Affymetrix, Santa Clara, CA) according to manufacture’s protocol.** Following RNA isolation, miRNA expression was measured using QuantiGene 2.0 Reagent System (Affymetrix) according to manufacture’s protocol. To capture mir-126 from samples, the capture plates containing samples and working probe set (capture extender (CE), label extender (LE), blocking probe (BL)) were incubated overnight at 55°C±1°C for hybridization. After hybridization using the Pre-Amplifier and Amplifer, the capture plate was hybridized with the label probe according to manufacture’s protocol. Luminescence was measured using a microplate luminometer after adding of 2.0 Substrate according to manufacture’s protocol. (The number of each group is n = 4.).
(TIFF)

Figure S4 **The purification of endothelial cells from Matrigel-embedded mouse eye tissue.** *A,* Mouse eye cups of each group were cultured for 3 days after embedding in Matrigel. At 3 days after culturing, each lysate was extracted from the Matrigel-embedded eye tissue (A) and the isolated endothelial cells using anti-mouse CD31 antibody-coated magnetuc beads (B). The amounts of CD31 and α-SMA were examined by Western blotting. *B,* Densitometry of α-SMA in panel A. *ANOVA* Statistical analysis performed. (n = 3) *, P<0.01. C,D, Mouse eye cups of each group were treated with EGFL7 or control siRNA after embedding them in Matrigel. Samples were cultured in VEGF (25 ng/ml) containing medium. At 3, 5, and 7 days after knockdown of EGFL7, endothelial cells were collected using anti-mouse CD31 antibody-coated magnetic beads. The purification of isolated endothelial cells was evaluated by qRT-PCR. The expression of α-SMA and CD31 mRNA in control, control siRNA and EGFL7 siRNA treatment groups were examined by qRT-PCR in panel C and D, respectively. ANOVA Statistical analysis performed to evaluate mRNA of αSMA. *, P<0.01.
(TIFF)
