# Supplementary figures and images for: EGF-Like-Domain-7 Is Required for VEGF-Induced Akt/ERK Activation and Vascular Tube Formation in an Ex Vivo Angiogenesis Assay
Source: PLoS One. 2014 Mar 19;9(3):e91849. doi: 10.1371/journal.pone.0091849 (PMC3960138; doi:10.1371/journal.pone.0091849)

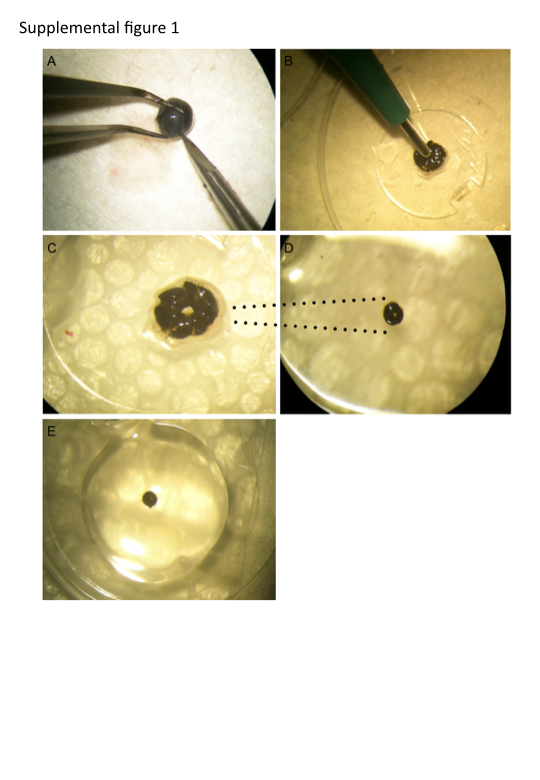

Supplement: Figure S1 — The creation of mouse eye cup and Histologic characterization of mouse eye cup embedded in Matrigel. After the removal of mouse cornea and lens (A), the eye sample (which contains retina-RPE-choroid-sclera) was excised in a circle round the optic nerve head using 1.0-mm skin biopsy punch (B,C and D), and embedded it into Matrigel (E). Schema of posterior segment of the eye that is used (F). Endothelial cells on the frozen mouse eye tissue section were immunostained with anti-CD31 antibody (G.H). The colour was developed using HRP conjugated secondary antibody and DAB staining. The section was then counterstained with methyl green. (TIFF) [file pone.0091849.s001.tiff]

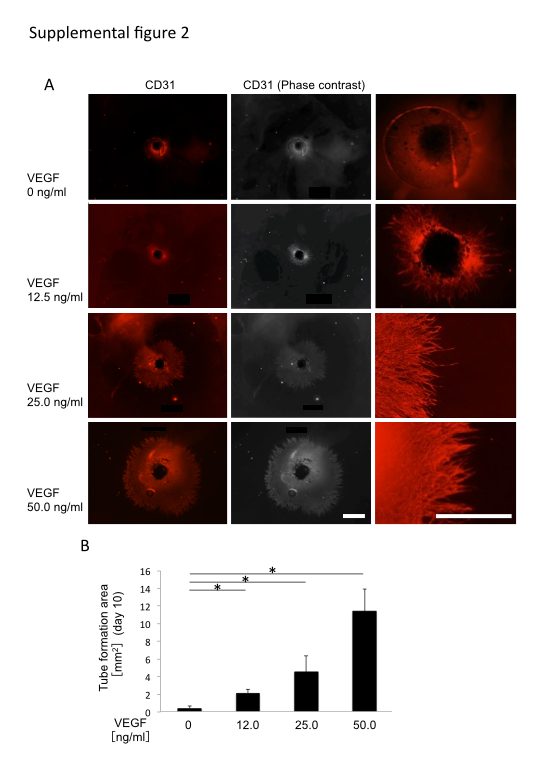

Supplement: Figure S2 — Dose and time dependent VEGF induction of neovascular tube formation from mouse eye cups. A, After the eye cups were embedded in Matrigel, each concentration of VEGF (0, 12.5, 25.0, 50.0 ng/ml) was added in the medium. At 10 days after culturing in these concentrations of VEGF-containing medium, the area of neovascularization from samples was evaluated by immunofluorescence using CD31 antibody. Medium was changed at days 3 and 7 after embedding. Bar equals 1000 μm. B, ANOVA Statistical analysis performed to evaluate the area of tube length (n = 6). *, P<0.01. C, After the eye tissue samples were embedded in Matrigel, tissue samples were cultured in medium containing 25.0 ng/ml VEGF for 3, 7, or 10 days,. At each day after culturing in VEGF-containing medium, the area of neovascular from samples was evaluated by immunofluorescence using CD31 antibody. Medium was changed at day 3 and 7 after embedding. Bar equals 1000 μm. D, ANOVA Statistical analysis performed to evaluate the area of tube length (n = 6). *, P<0.01. **, P<0.05. (TIFF) [file pone.0091849.s002.tiff]

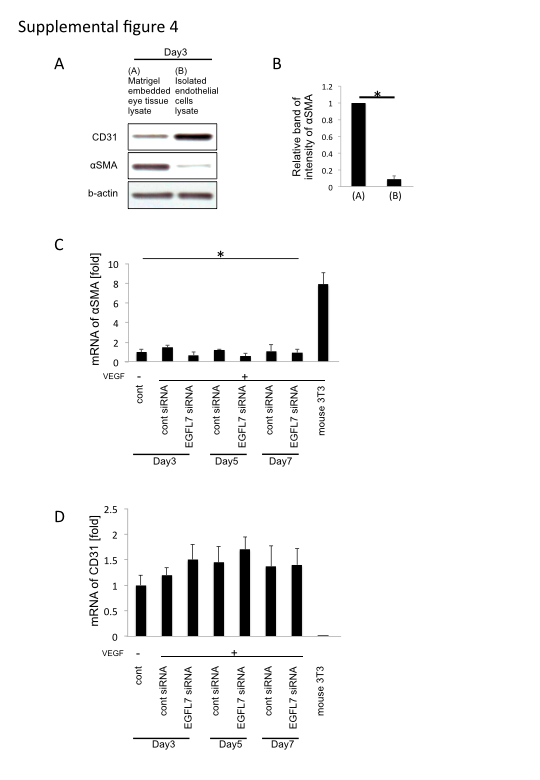

Supplement: Figure S4 — The purification of endothelial cells from Matrigel-embedded mouse eye tissue. A, Mouse eye cups of each group were cultured for 3 days after embedding in Matrigel. At 3 days after culturing, each lysate was extracted from the Matrigel-embedded eye tissue (A) and the isolated endothelial cells using anti-mouse CD31 antibody-coated magnetuc beads (B). The amounts of CD31 and α-SMA were examined by Western blotting. B, Densitometry of α-SMA in panel A. ANOVA Statistical analysis performed. (n = 3) *, P<0.01. C,D, Mouse eye cups of each group were treated with EGFL7 or control siRNA after embedding them in Matrigel. Samples were cultured in VEGF (25 ng/ml) containing medium. At 3, 5, and 7 days after knockdown of EGFL7, endothelial cells were collected using anti-mouse CD31 antibody-coated magnetic beads. The purification of isolated endothelial cells was evaluated by qRT-PCR. The expression of α-SMA and CD31 mRNA in control, control siRNA and EGFL7 siRNA treatment groups were examined by qRT-PCR in panel C and D, respectively. ANOVA Statistical analysis performed to evaluate mRNA of αSMA. *, P<0.01. (TIFF) [file pone.0091849.s004.tiff]
